# No evidence for superior distractor filtering amongst individuals high in autistic-like traits

**DOI:** 10.3758/s13414-022-02575-3

**Published:** 2022-10-07

**Authors:** Troy A. W. Visser, Michael C. W. English, Murray T. Maybery

**Affiliations:** School of Psychological Science, University of Western Australia, 35 Stirling Hwy, Crawley, WA 6009 Australia

**Keywords:** Visual search, Attention, Attentional blink

## Abstract

Autistic individuals and individuals with high levels of autistic-like traits often show better visual search performance than their neurotypical peers. The present work investigates whether this advantage stems from increased ability to filter out distractors. Participants with high or low levels of autistic-like traits completed an attentional blink task in which trials varied in target-distractor similarity. The results showed no evidence that high levels of autistic-like traits were associated with superior distractor filtering (indexed by the difference in the size of the attentional blink across the high- and low-similarity distractors). This suggests that search advantages seen in previous studies are likely linked to other mechanisms such as enhanced pre-attentive scene processing, better decision making, or more efficient response selection.

Autism spectrum disorder^1^ is characterized by broad difficulties in social functioning and communication, along with restricted interests and behaviours (DSM-5, American Psychiatric Association, 2013). However, while these behavioural attributes are central to the definition of the condition, many researchers, beginning with Kanner (1943), have also documented consistent links between autism and atypical perceptual processing (e.g., Behrmann et al., 2006; Mottron et al., 2003; Simmons et al., 2009; Wang et al., 2007). Moreover, atypical perception has been increasingly linked to social functioning (e.g., English et al., 2015; Frith, 1989; Mottron & Burack, 2001), suggesting that a better understanding of atypical perception may also yield insights into the origins of atypical social and behavioural attributes associated with autism.

Perhaps the most frequently discussed perceptual processing difference between autistic and non-autistic individuals involves global and local processing. When presented with a hierarchical figure consisting of a larger object made up of smaller features (e.g., the letter A composed of smaller letter Ts; Navon, 1977), autistic individuals are typically quicker and more accurate at perceiving the smaller (local) features than their non-autistic peers (e.g., Behrmann et al., 2006; Muth et al., 2014; Pellicano et al., 2006; but see Van der Hallen et al., 2015). However, these are not the only perceptual differences that have been documented. Studies have also suggested that autistic and non-autistic individuals differ in the magnitude of left visual field bias (Wainwright & Bryson, 1996), the impact of cues on spatial orienting (Wainwright-Sharp & Bryson, 1993), and visual search efficiency (Joseph et al., 2009; Kemner et al., 2008; O’Riordan et al., 2001; O’Riordan, 2004).

The perceptual differences found between autistic and non-autistic individuals are mirrored in neurotypical individuals who differ in levels of autistic-like traits (ALTs), as assessed using measures such as the Autism Spectrum Quotient (AQ; Baron-Cohen et al., 2001). For example, like Wainwright and Bryson (1996), English et al. (2015, 2017a) showed a smaller left visual field bias for high-ALT individuals than low-ALT individuals using a greyscales task (Nicholls et al., 1999) in which individuals had to judge which of two shaded bars was “darker”. Similarly, Almeida and colleagues (Almeida et al., 2010a, 2010b; Almeida et al., 2013) showed robust evidence for superior visual search in high-ALT individuals compared to their low-ALT peers (for other examples, see Bayliss & Kritikos, 2011; Cribb et al., 2016; Grinter et al., 2009; although see Gregory & Plaisted-Grant, 2016, and Pérez et al., 2019, for contrary results). These parallel findings in autistic and high-ALT groups are consistent with the notion that ALTs are normally distributed in the general population, extending out to extremes required for clinical diagnosis (e.g., Ronald & Hoekstra, 2011; Skuse et al., 2005; Sucksmith et al., 2011).

The focus of the present work is on investigating the mechanisms that underlie perceptual advantages seen in autistic and high-ALT individuals in visual search tasks that require finding a target presented amongst non-target distractors. At present, several alternative mechanisms have been put forward. Frith (1989) and others have suggested that perceptual advantages might be linked to local processing biases that enhance stimulus processing. Others have suggested that search benefits might reflect superior ability to distinguish between target and non-target features, thereby enhancing stimulus discriminability and allowing resources to be more efficiently allocated to targets (Kemner et al., 2008; O’Riordan & Plaisted, 2001).

Here, we investigate a third option suggested by Joseph et al. (2009), who compared performance between children and adolescents with and without autism in a dynamic visual search task in which search items were re-plotted at new locations every 500 ms. Joseph et al. (2009) found significantly lower search intercepts in the autistic group, which they speculated might be due to autistic individuals being faster at processing items at the focus of attention, thereby allowing quicker target identification, and presumably also faster identification and rejection of non-target distractors. Thus, on this account, superior target processing may arise not only from faster target processing as suggested by O’Riordan and Plaisted (2001), but also from more efficient rejection of distractors.

The notion that autistic and high-ALT individuals might be better at rejecting distractors was also suggested by Spaniol et al. (2018), who asked university undergraduate individuals with varying levels of ALTs to complete two tasks. One was a Navon task in which individuals had to identify global or local letters across different blocks of trials. The other task had individuals identify either faces or scenes that were superimposed on each other and mapped to the same or different response keys. Critically, compared to low-ALT individuals, high-ALT individuals showed a smaller incongruency effect in the Navon task (i.e., were less adversely affected when the global and local level stimuli differed), and demonstrated better performance in the other task when response interference was greatest (i.e., when the face and scene were mapped to the same response key). Based on these results, Spaniol et al. (2018) concluded that high-ALT individuals were better able to filter out distractors than their low-ALT counterparts, particularly when the task was more challenging.

Notably, while the results of Joseph et al. (2009) and Spaniol et al. (2018) support the notion that autism/ALT is associated with superior distractor filtering, several alternative explanations are also possible. In the case of Joseph et al. (2009), as the authors point out, variations in search intercepts could be due to differences in pre-attentive perceptual processes and/or perceptual decision making, rather than more efficient distraction rejection. There is also evidence that distractor processing can interfere with shifts of spatial attention (Folk et al., 2008; Visser, 2011), thus opening up the possibility that differences in autistic and non-autistic individuals reflect more efficient spatial shifting to items spread across the search display. In the case of Spaniol et al. (2018), results might reflect the small number of target and distractor stimuli used in their tasks, and the fact that they remained constant across blocks of trials. If this were the case, it is possible that advantages for autistic or high-ALT individuals might only occur when stimulus and response options are highly constrained. Lastly, because both experiments required speeded responses, it is possible that differences across individual groups reflect variations in response selection efficiency rather than perceptual filtering.

To rule out these alternative explanations, we compared distractor interference across participants with differing levels of ALTs using an attentional blink (AB) paradigm in which participants viewed a series of stimuli presented at a centrally fixated location. Participants were asked to report the identity of two letter targets (T1 and T2 respectively), separated by a variable temporal interval (lag), embedded amongst multiple distractors within this stream. These distractors varied such that they were similar (pseudoletters) or dissimilar (keyboard symbols) to the letter targets. Targets and distractors were chosen randomly on each trial and drawn from a large stimulus set to avoid constraining stimulus and response options. Additionally, identification responses were unspeeded and made at the end of the stimulus sequence to minimize response selection demands.

Multiple AB studies have shown that identification of T1 is highly accurate and invariant with lag, while identification of T2 shows an “attentional blink” with impaired performance at short lags and gradual improvement to the level of T1 accuracy by a lag of roughly 500 ms (Chun & Potter, 1995; Raymond et al., 1992; Visser & Ohan, 2007; see Dux & Marois, 2009, for a review). Conventional accounts attribute the AB to selection and high-level processing of T1, which interferes with T2 processing when the two targets are presented in close temporal proximity (e.g., Chun & Potter, 1995; Shapiro & Raymond, 1994; Visser et al., 1999; Taatgen et al., 2009).

Importantly, several studies have also shown that the AB is modulated by target-distractor similarity (e.g., Chun & Potter, 1995; Folk et al., 2008; Ghorashi et al., 2003; Jolicœur et al., 2006; Tang et al., 2020). For example, Visser et al. (2004) found that the magnitude of the AB for letter targets increased as target-distractor similarity increased from low (e.g., random-dot patch distractors) to high (e.g., pseudoletters composed of scrambled letter features) levels. They explained this phenomenon in the context of existing AB models, suggesting that distractors that shared target features sometimes were errantly selected as targets, thereby preventing resources from being allocated to T2. Put differently, they suggested distractor interference arose from a failure to adequately filter out high-similarity distractors. This account is akin to explanations for other distractor-related perceptual impairments such as inattentional blindness (Mack & Rock, 1998), surprise capture (Horstmann & Ansorge, 2016), and emotion-induced blindness (Wang et al., 2012).

There are several advantages to using the AB paradigm to study distractor suppression compared to the visual search paradigm. As noted above, it avoids taxing response selection processes that may differ between neurotypical individuals and those with autism/high-ALT. Further, unlike visual search tasks, shifts of spatial attention or eye movements are not required to do the task as all stimuli are presented at a known, central spatial location that is fixated. This avoids potential confounds that could arise from differences in the size of the focus of spatial attention (Robertson et al., 2013) or in the speed of shifting visual attention (Ronconi et al., 2018) that have been documented between neurotypical individuals and those with autism/high-ALT.

Moreover, the AB and visual search also tap similar mechanisms and have overlapping neural underpinnings. For example, in both phenomena, search for targets amongst distractors is guided by target templates (Chun & Potter, 1995; Ghorashi et al., 2003; Wolfe, 2007, 2020) that are compared to incoming stimuli to separate targets from distractors based on distinguishing features. This suggests that evidence concerning distractor processing gleaned from the AB is likely to generalize to visual search. In addition, studies indicate spatial neglect patients also show an abnormally prolonged AB (Hillstrom et al., 2004; Husain et al., 1997). This suggests that spatial shifts of attention required for search and shifts of temporal attention required during the AB share at least some common neural substrates.

Previous work has suggested that the magnitude of the AB deficit for neutral-valence stimuli is largely equivalent across neurotypical, autistic and high-ALT of all ages (Amirault et al., 2009; English et al., 2017b, 2019; Gaigg & Bowler, 2009;Rinehart et al., 2010 ; Yerys et al., 2013). These results were obtained using AB paradigms that consisted of a single stream of centrally presented targets and distractors. Streams contained a variety of target stimuli and associated tasks, including letter detection/identification (Amirault et al., 2009; Rinehart et al., 2010), word identification (Gaigg & Bowler, 2009), and face detection (English et al., 2017b, 2019; Yerys et al., 2013). Participants also variously included adults aged 18–36 years (Amirault et al., 2009; English et al., 2017b, 2019; Gaigg & Bowler, 2009) and children aged 8–14 years (Rinehart et al., 2010; Yerys et al., 2013).

Importantly, however, no previous study has investigated the impact of varying target-distractor similarity on the AB and how this might vary with levels of ALTs. That said, the studies reviewed above suggest clear predictions about how high- and low-ALT individuals would perform in an AB paradigm with varying levels of target-distractor similarity. Low-ALT individuals should show a larger AB when target-distractor similarity is high than when it is low. By comparison, if high-ALT individuals are better able to filter out distractors as posited by Spaniol et al. (2018), then they should show little or no change in AB magnitude when target-distractor similarity is high compared to when it is low. On the other hand, if high-ALT individuals are not better able to filter out distractors, then they should show approximately the same increase in AB magnitude when target-distractor similarity is increased as their low-ALT peers.

## Methods

### Participants

Participants were a convenience sample of 229 students enrolled in an undergraduate psychological research methods and statistics unit at a university. They completed the experiment as part of a classroom activity at individual workstations in groups of 18–20. Participants completed the task by themselves and were asked to maintain quiet focus on their work. Four participants, whose overall T1 accuracy was more than 3 standard deviations below the group mean, were identified as outliers, and not considered in the data analysis reported below. Thus, the final sample consisted of 225 students (56 males, 167 females, two did not respond) with a mean age of 21.64 years (SD = 5.91). Approval to conduct the study was received from the Human Research Ethics Office at the university and the study was carried out in accordance with the provisions of the World Medical Association Declaration of Helsinki. All participants provided written informed consent prior to participation.

### Materials

#### Questionnaire

Autistic traits were assessed using the 50-item self-report Autism Spectrum Quotient (AQ; Baron-Cohen et al., 2001). The questionnaire uses a four-item forced-choice format, and scoring was done using the 1–4 method introduced by Austin (2005), with higher scores indicating greater levels of autistic traits. The scoring method proposed by Austin (2005) takes advantage of the full range of potentially useful information in each item, thus increasing the variability of total AQ scores (Stevenson & Hart, 2017). Moreover, it yields similar internal consistency (.82: Austin, 2005) to Baron-Cohen et al.’s binary scoring method (.67: Hurst et al., 2007; .79: Freeth et al., 2013).

#### Attentional blink task

Each participant was seated approximately 50 cm in front of a 23.8-in. Dell P2419H monitor display running at 1,920 × 1,080 resolution and connected to a computer running Windows 10. Presentation software (Ver 20.1, Neurobehavioral Systems) was used to generate and display task stimuli and record participant responses from standard QWERTY-layout keyboards. Targets consisted of all possible letters presented in upper case Arial 32-point font, except for I, O, P, Q and Z as these letters are easily confused with digits. Targets were embedded among distractor stimuli that consisted of pseudoletters (high target-distractor similarity) or keyboard symbols (low target-distractor similarity). Pseudoletters were drawn from a set of 19 stimuli (see Visser, 2007), while symbols consisted of #, @, ?, % and &. Each target was masked by a digit (0–9) on the assumption that masks that were more similar to targets would be more effective. A digit mask was used on all trials to avoid confounding changes in overall target-distractor similarity with the effectiveness of the target masks. Targets, masks and distractor stimuli subtended approximately 1.0° × 1.0° of visual angle and were presented in medium grey (RGB: 167, 167, 167) on a black background (RGB: 0, 0, 0). Note that we cannot report exact stimulus luminance due to variability across workstations.

### Procedure

Instructions for all tasks and measures were displayed on-screen prior to commencement, and participants were able to ask researchers for clarification at any point. Each experimental session commenced with the AB task, followed by completion of the AQ and demographics questionnaires.

**Fig. 1:**
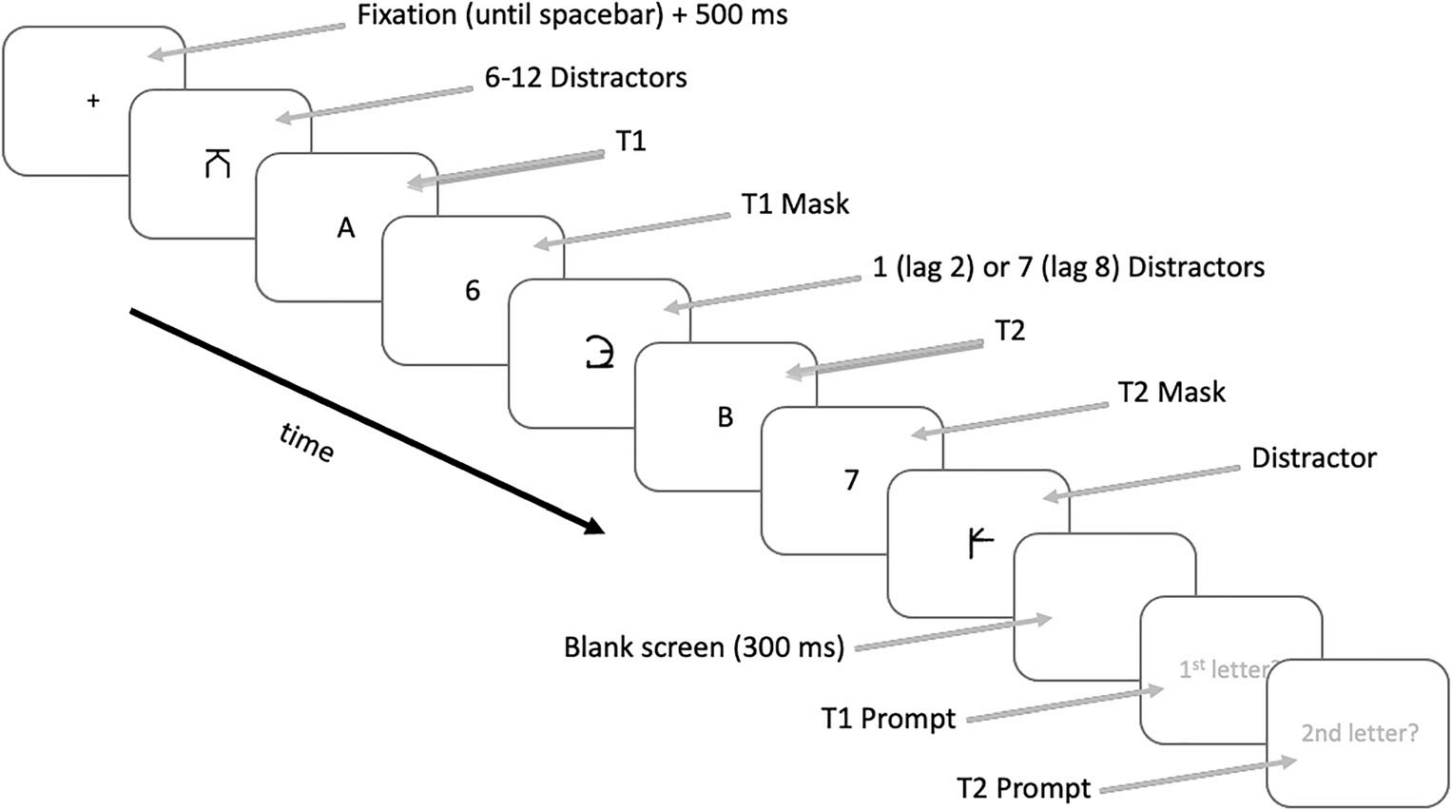
Schematic presentation of a typical trial in the AB task (not to scale). The figure depicts a high-similarity trial with pseudoletter distractors

A typical trial on the AB task is illustrated in Fig. 1. Each trial began with the presentation of a fixation cross in the centre of the screen. Participants were asked to press the space bar to begin the trial once they had fixated on this cross. Once the trial was initiated, the fixation cross remained on the display for 500 ms, and then the stimulus sequence commenced with the presentation of 6–12 distractors (number varied randomly across trials), followed by T1, and the T1 mask. The T1 mask was followed by one (lag 2) or seven (lag 8) additional distractors, and then T2, the T2 mask, and a final distractor. Each of these items was presented for 67 ms with a 0-ms inter-stimulus interval. Following a 300-ms blank display, participants were then prompted to enter each of the letters in the stream in the order that they appeared and were instructed to guess if they were unsure.

Distractors were chosen randomly with replacement from the set of either pseudoletters or keyboard symbols (with the constraint that the same distractor could not be presented consecutively), while masks were chosen randomly from the set of digits. The AB task was presented in two blocks of 64 trials divided evenly across all possible combinations of lag and target-distractor similarity and presented in random order to prevent anticipation of distractor type or lag. Participants were encouraged to take a rest break when needed when the fixation cross was on the display, and between the two blocks of trials.

In addition to the requirement to identify two targets on each trial, participants also completed a face emotion matching task as part of a separate study. For this task, participants viewed a face prior to and following each AB trial and were asked to report whether the face depicted the same or different emotions across the two presentations. The faces were unrelated to the stimuli or responses in the main AB task, and analyses indicated emotion identification was unrelated to any of the AB results reported below.

## Results

Although participants were prompted to enter target identity in the order they were presented, following the analysis approach of our previous studies (e.g., English et al., 2017b; Visser et al., 2004), target accuracy on the AB task was calculated without consideration of response order to maximize statistical power by increasing the number of trials on which conditional T2 accuracy could be calculated (see description of T2|T1 calculations below). ALT comparison groups were created by selecting participants in the top and bottom 20% of the distribution of AQ scores. We chose this extreme-groups analytical approach over a regression analysis on the grounds that there is evidence it increases statistical power (Cribb et al., 2016).

The Low AQ group consisted of participants with an AQ score ≤ 96 (n = 47), while the high AQ group consisted of participants with an AQ score ≥ 120 (n = 57). These AQ cutoff values are comparable to those used by English et al. (2017b) – low AQ ≤ 96; high AQ ≥ 116 – and more extreme than those used by English et al. (2019) – low AQ ≤ 105; high AQ > 105. With these groups, our analyses had an a priori power of approximately 0.90 at an alpha level of. .05, even when assuming a small effect size of 0.13 (Faul et al., 2007). Mean AQ scores were substantially lower in the Low AQ group (mean = 90.49, range = 68–96, SD = 6.21) than in the high AQ group (mean = 128.20, range = 120–180), SD = 11.94), *t*(101) = 19.53, *p* < .001. However, the mean age of the Low AQ group (mean = 21.83, range = 18–47, SD = 6.96) did not differ significantly from the mean age of the High AQ group (mean = 22.49, range = 18–49, SD = 6.50), *t*(100) = 0.50, *p* > .62. The sex ratio also did not vary between the Low AQ group (36 female, ten male, one prefer not to say) and the High AQ group (41 female, 14 male, one prefer not to say), *x*^*2*^(2) = 0.21, *p* > .90.

### T1 accuracy

Mean T1 accuracy scores, calculated separately as a function of target-distractor similarity (high vs. low), lag (2 vs. 8), and AQ group (low vs. high), are presented in Table 1. These means were submitted to a Similarity × Lag × AQ group repeated-measures analysis of variance (ANOVA). This analysis yielded a significant main effect of Similarity, F(1, 101) = 98.77, p < .001, n^2^_p_ = .494. Examination of Table 1 suggests that this reflects clearly poorer T1 performance when target-distractor similarity was high compared to when it was low. The main effects of Lag [F(1, 101) = 0.51, p = .48, n^2^_p_ < .02] and AQ group [F(1, 101) = 3.25, p = .07, n^2^_p_ = .03], as well as the Lag × AQ group [F(1, 101) = 0.06, p = .80, n^2^_p_ < .01], Similarity × AQ group [F(1, 101) = 0.39, p = .53, n^2^_p_ < .01], Similarity × Lag [F(1, 101) = 0.52, p = .47, n^2^_p_ < .02], and Similarity × Lag × AQ Group [F(1, 101) = 0.03, p = .86, n^2^_p_ < .01] interactions were all non-significant.

**Table 1:** Mean T1 identification accuracy as a function of distractor type (low vs. high target similarity), inter-target lag (2 vs. 8), and AQ group (Low vs. High)

	Distractor type	Low similarity		High similarity	
	Inter-target lag	2	8	2	8
AQ Group	Low	86.2 (1.51)	85.0 (1.75)	72.7 (2.33)	72.6 (2.32)
	High	89.0 (1.28)	88.4 (1.25)	77.3 (2.20)	77.4 (2.01)

Numbers in parentheses represent one standard error of the mean

To more robustly assess whether there was any evidence that AQ group modulated the impact of target-distractor similarity on T1 performance, we also examined the data using a Bayesian ANOVA. Bayes factors were calculated with *jamovi*, using a default Cauchy prior width of *r* = 0.707. The null hypothesis was that there were no target accuracy differences across levels of individual factors (e.g., Lag) or interactions between levels of those factors (e.g., Lag and AQ group). The alternative hypothesis was that there were target accuracy differences across levels of individual factors or interactions between levels of those factors. As can be seen in Table 2, there was decisive evidence (Jeffreys, 1961) in favour of the alternative hypothesis, indicating Similarity influenced T1 accuracy. However, there was substantial evidence (Jeffreys, 1961) in favour of the null hypothesis for the Similarity × AQ Group and Lag × Similarity × AQ group interactions.

**Table 2:** Outcomes of Bayesian ANOVA conducted on T1 accuracy scores

Effect	BF_10_
Lag	0.12
Similarity	5.94 × 10^30^
AQ Group	0.89
Lag × Similarity	0.19
Lag × AQ Group	0.16
Similarity × AQ Group	0.20
Lag × Similarity × AQ Group	0.21

BF_10_ scores estimate amount of evidence in favour of the alternative hypotheses: scores greater than 100 indicate extreme evidence in favour of the alternative hypothesis, scores between .33 and 1 indicate anecdotal evidence for the null hypothesis, and scores between .10 and .33 indicate moderate evidence for the null hypothesis (Jeffreys, 1961)

### T2|T1 accuracy

**Fig. 2:**
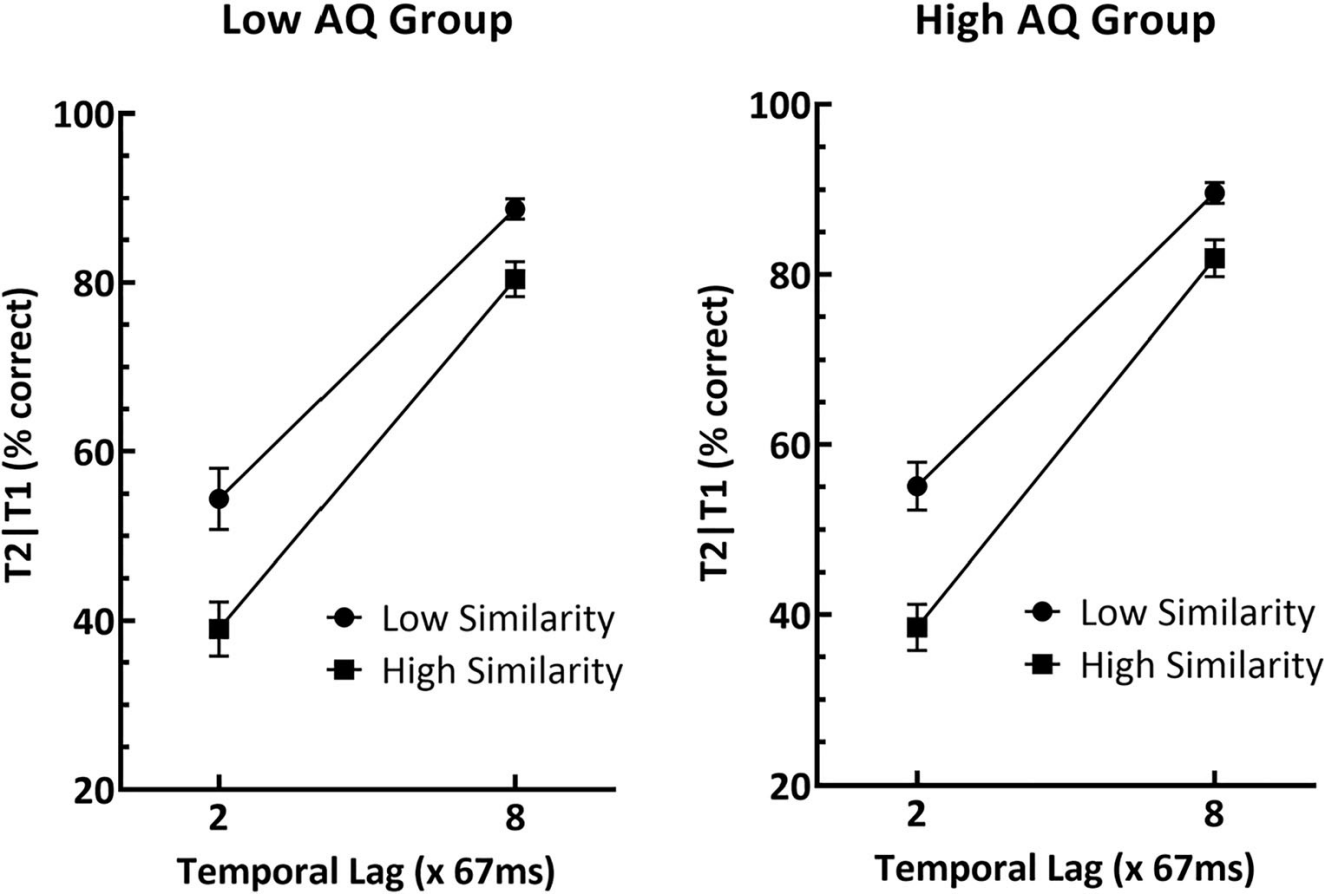
T2|T1 accuracy as a function of target-distractor similarity and temporal lag. The left graph depicts performance in the Low AQ group. The right graph depicts performance in the High AQ group. Error bars represent one standard error of the mean

Mean T2 accuracy was calculated only on trials in which T1 was identified correctly on the grounds that the source of T2 errors on other trials is unknown (Raymond et al., 1992). These scores, separated as a function of target-distractor similarity, lag, and AQ group, are shown in Fig. 2, and were submitted to a Similarity × Lag × AQ group repeated-measures ANOVA. This analysis yielded a significant main effect of Lag, F(1, 101) = 539.43, p < .001, n^2^_p_ = .842, and Similarity, F(1, 101) = 84.55, p < .001, n^2^_p_ = .456. Consistent with previous studies that examined the impact of target-distractor similarity on the AB (e.g., Visser et al., 2004), there was also a significant Lag × Similarity interaction, F(1, 101) = 19.55, p < .001, n^2^_p_ = .162, indicating that the AB was more pronounced when similarity was higher. Critically, however, there was no AQ Group × Similarity interaction, F(1, 101) = 0.009, p = .93, n^2^_p_ < .001, nor the AQ Group × Similarity × Lag interaction, F(1, 101) = 0.24, p = .62, n^2^_p_ = .002, that would be expected if high-ALT individuals were better able to ignore distractors. All other main effects and interactions were also non-significant (F < .13, p > .72, n^2^_p_ = .001).

As with the T1 accuracy results, we also examined the data using a Bayesian ANOVA. As can be seen in Table 3, the results yielded decisive evidence in favour of the alternative hypothesis for main effects of Lag and Similarity, as well as substantial evidence in favour of the alternative hypothesis for the Lag × Similarity interaction (Jeffreys, 1961). Critically, however, there was substantial evidence in favour of the null hypothesis for the AQ × Similarity and AQ × Lag × Similarity interactions (Jeffreys, 1961). In sum, the results robustly indicate that target-distractor similarity interacted with the AB, as in previous studies, but that this interaction was not moderated by level of ALTs.

**Table 3:** Outcomes of Bayesian ANOVA conducted on T2|T1 accuracy scores

Effect	BF_10_
Lag	1.26 × 10^85^
Similarity	6.08 × 10^13^
AQ Group	0.27
Lag × Similarity	9.35
Lag × AQ Group	0.15
Similarity × AQ Group	0.15
Lag × Similarity × AQ Group	0.23

BF_10_ scores estimate amount of evidence in favour of the alternative hypothesis: scores greater than 100 indicate extreme evidence in favour of the alternative hypothesis, scores between 3 and 10 indicate moderate evidence for the alternative hypothesis, and scores between .10 and .33 indicate moderate evidence for the null hypothesis (Jeffreys, 1961)

## Discussion

While autism and high levels of ALTs are often strongly associated with atypical social-emotional processing, increasing evidence has shown that they are also linked to alterations in perceptual processing. Here we investigated the origins of the well-documented advantages in search task performance often observed in autistic and high-ALT individuals (Almeida et al., 2013; Frith, 1989; Kemner et al., 2008; O’Riordan & Plaisted, 2001). Specifically, we tested whether high-ALT individuals might be superior at filtering out distracting stimuli (Spaniol et al., 2018) compared to their low-ALT peers. To test this possibility, we asked participants with varying levels of ALTs (as assessed by the AQ; Baron-Cohen et al., 2001) to complete an AB task that presented targets along with high- or low-similarity distractors. Past studies have shown that increasing target-distractor similarity leads to a larger AB (e.g., Ghorashi et al., 2003; Visser et al., 2004), which has been suggested to reflect an inability to filter out high-similarity distractors (Di Lollo et al., 2005; Visser et al., 2004). Thus, we reasoned that if high-ALT participants were better able to filter out distractors, they would show less of a performance decrement when target-distractor similarity was high compared to low-ALT participants.

Consistent with past work, we found that increased target-distractor similarity led to a greater decrement in T2 accuracy (i.e., a larger AB). However, this effect was not modulated by level of ALTs. There were no significant interactions involving AQ group and target-distractor similarity, and evidence from supplementary Bayesian ANOVAs was strongly in favour of the null hypothesis for these interactions. This suggests that, at least in the context of the AB paradigm, high-ALT individuals are not better at filtering out distractors that are similar to targets, and thus suffer detriments to target identification analogous to those experienced by low-ALT individuals.

One possible explanation for the discrepant outcomes between our experiment and past studies (Joseph et al., 2009; Spaniol et al., 2018) is our use of the AB paradigm. However, while we cannot rule out this possibility based on our current data, we think it is unlikely because our paradigm possessed broadly similar characteristics to AB tasks used in previous work. Like Spaniol et al. (2018), our stimuli were presented centrally at fixation limiting the need for eye movements, and like Joseph et al. (2009), our displays changed rapidly over the course of an experimental trial. Rather than paradigmatic differences, we suggest that the most likely explanation for the differences between our results and those of Spaniol et al. (2018) stems from the differing number of distractor and target options.

Whereas distractors and targets in our paradigm were chosen randomly from large sets of options and varied across trials, Spaniol et al. (2018) drew targets and distractors from a small pool of items and their identities remained constant across blocks of trials. These stimulus and response constraints likely assisted in the development of better distractor filtering. By comparison, the unpredictable stimulus streams in our AB paradigm prevented the establishment of consistent filters and predictable responses, thereby eliminating advantages for high-ALT participants.

This explanation also accords well with predictive coding accounts of autism (Lawson et al., 2014; Sinha et al., 2014; Van de Cruys et al., 2014). For example, Van de Cruys et al. (2014) suggested that the precision of predictions about future events derived from past experience (i.e., predictive coding) is at least as good, and perhaps better in some instances, for individuals with autism than for non-autistic individuals. However, individuals with autism are less able to isolate task-relevant predictive relationships in complex environments, thus leading to performance decrements in these situations. Following this logic, it might be expected that the more predictable stimulus presentation environment in Spaniol et al. (2018) would better support predictive coding in high-ALT individuals, compared to the more complex and varied stimulus presentation conditions employed here. In turn, this might be expected to yield an advantage for high-ALT individuals in Spaniol et al. (2018) that would not be present in our study.

Some suggestive evidence in favour of these explanations comes from Ghorashi et al. (2003), who found that the impact of high-similarity distractors on the AB disappeared when the same distractor was presented repeatedly across trials. This outcome implies that having a predictable distractor can significantly improve filtering, but only under very limited circumstances in unusually predictable environments. It is also notable that Van der Hallen et al. (2017) found no differences between autistic and neurotypical children in global or local interference when performing a hierarchical letters task with high levels of stimulus uncertainty. As with our findings, Van der Hallen et al. (2017) suggested that their failure to find group differences might reflect the fact that their unpredictable stimuli prevented autistic individuals from developing perceptual and response strategies to support performance.

While the present experiment focused primarily on the importance of target-distractor similarity effects on the AB, it is worth noting that we also found that T1 accuracy was impaired by increasing target-distractor similarity. Such an outcome is not unusual (e.g., Visser et al., 2009), and suggests that high-similarity distractors can be errantly selected even when cognitive resources are not already occupied with processing other target stimuli as is the case in the AB. However, as was the case with T2 accuracy, we found no evidence that distractor effects on T1 were ameliorated for individuals with high levels of ALTs. Thus, it appears that high-ALT individuals show no advantages at filtering out distractors either when cognitive resources are depleted (i.e., the AB), or when they are fully available for stimulus processing (i.e., T1).

While the present findings do not support the option that high-ALT individuals are better at filtering out distractors, this in no way takes away from the significant body of literature that suggests autistic and high-ALT individuals are superior at visual search for targets amongst distractors. Given our findings, however, we suggest that advantages in visual search seen in these studies could be explained by other aspects of distractor-related processing. Some of the possible explanations are suggested by Joseph et al. (2009), who noted that the intercept differences between autistic and non-autistic individuals in their search task could reflect variations in pre-attentive perceptual processes and/or perceptual decision making. As noted above, it is also possible that under highly constrained conditions such as those present in Spaniol et al. (2018), high-ALT individuals could be better at filtering out distractors, but that this advantage disappears in more varied environments. Finally, in cases where tasks emphasize search speed (e.g., Joseph et al., 2009), superior performance in autistic and high-ALT individuals might reflect differences in response selection mechanisms that were not tapped in our unspeeded AB task. At present, all of these alternatives are plausible. Thus, there is a clear need for systematic work in future studies to better understand the mechanisms underlying search advantages linked to autism and high levels of ALTs.

Finally, several potential limitations of our study should also be noted. First, our participant sample consisted of a high proportion of female undergraduate university students who were studying psychology. This may limit the comparability of our findings with previous research that has employed more predominantly male participant groups with autism, as well as the generalizability of our work to non-university samples. A second consideration is whether our high- and low-AQ groups were sufficiently differentiated to detect performance differences related to ALT. While we cannot exclude the possibility that ALT-related differences could emerge with more disparate ALT groups, it is notable that differences in emotional guidance of attention in an AB paradigm were detected using similar (English et al., 2017b) and less differentiated (English et al., 2019) samples. Third, it is possible that distractions arising from ambient noise in the group testing environment or working memory load imposed by the requirement to withhold target responses until the end of a trial might have differentially affected our low- and high-ALT groups. However, we believe such putative differences are unlikely to explain our findings as they would have influenced all conditions equally and because there is no evidence that the ALT groups performed differently on target identification overall (i.e., no main effects/interactions of AQ for T1 or T2).

In summary, the present results do not suggest that individuals with high levels of ALTs are better able to filter out distracting stimuli that share target features than their peers with lower levels of ALTs. This implies that documented advantages in visual search tasks (e.g., Almeida et al., 2013; Joseph et al., 2009) in autistic and high-ALT individuals stem from other advantages that may be tied to pre-attentive processing, decision processes, or, in some cases, response selection. Given evidence for parallel task outcomes across autistic and high-ALT individuals, including in the visual search task and attentional blink paradigms (e.g., English et al., 2017b, 2019; Rinehart et al., 2010), we tentatively suggest that our findings here are likely to extend to autistic individuals. However, we cannot be sure of this in the absence of replicating our experiment with an autistic group. Thus, it will be critical for future studies to do so to ensure our results extend more broadly. An additional avenue for future work will be to verify the perceptual conditions that lead to improved search performance amongst autistic individuals or those with high levels of ALT to establish how these improvements might be linked to other phenomena such as social functioning.

## Footnotes

1 We use the term *Autism Spectrum Disorder* here in keeping with the Diagnostic and Statistical Manual of Mental Disorders, Fifth Edition, but have avoided ableist language elsewhere in the paper as per the guidelines of Bottema-Beutel et al. (2021).
